# Zebrafish larvae as experimental model to expedite the search for new biomarkers and treatments for neonatal sepsis

**DOI:** 10.1017/cts.2021.803

**Published:** 2021-06-18

**Authors:** Fleur M. Keij, Bjørn E. V. Koch, Fernando Lozano Vigario, Sinno H. P. Simons, Johan G. C. van Hasselt, H. Rob Taal, C. A. J. Knibbe, Herman P. Spaink, Irwin K. M. Reiss, Elke H. J. Krekels

**Affiliations:** 1 Erasmus MC Sophia Children’s Hospital, Erasmus University Medical Center Rotterdam, Rotterdam, the Netherlands; 2 Division of Animal Sciences and Health, Institute of Biology Leiden, Leiden University, Leiden, the Netherlands; 3 Division of Biotherapeutics, Leiden Academic Centre for Drug Research, Leiden University, Leiden, the Netherlands; 4 Division of Systems Biomedicine and Pharmacology, Leiden Academic Centre for Drug Research, Leiden University, Leiden, the Netherlands; 5 Department of Clinical Pharmacy, St Antonius Hospital, Nieuwegein, the Netherlands

**Keywords:** Neonatal sepsis, zebrafish larvae, preclinical disease model, biomarker discovery, drug development

## Abstract

Neonatal sepsis is a major cause of death and disability in newborns. Commonly used biomarkers for diagnosis and evaluation of treatment response lack sufficient sensitivity or specificity. Additionally, new targets to treat the dysregulated immune response are needed, as are methods to effectively screen drugs for these targets. Available research methods have hitherto not yielded the breakthroughs required to significantly improve disease outcomes, we therefore describe the potential of zebrafish (Danio rerio) larvae as preclinical model for neonatal sepsis. In biomedical research, zebrafish larvae combine the complexity of a whole organism with the convenience and high-throughput potential of *in vitro* methods. This paper illustrates that zebrafish exhibit an immune system that is remarkably similar to humans, both in terms of types of immune cells and signaling pathways. Moreover, the developmental state of the larval immune system is highly similar to human neonates. We provide examples of zebrafish larvae being used to study infections with pathogens commonly causing neonatal sepsis and discuss known limitations. We believe this species could expedite research into immune regulation during neonatal sepsis and may hold keys for the discovery of new biomarkers and novel treatment targets as well as for screening of targeted drug therapies.

## Introduction

Neonatal sepsis remains a major cause of mortality accounting for 15.6% of neonatal deaths worldwide [1]. Apart from mortality, it is associated with long-term consequences including impaired neurodevelopment [2]. A consensus definition for neonatal sepsis is lacking, but it is recognized as a systemic condition, with a dysregulated immune reaction in response to a pathogen, resulting in harmful hemodynamic changes and potential organ dysfunction [3,4].

Neonatal sepsis compromises different entities influenced by aspects such as the gestational and postnatal age of the patient and the source of infection. It is traditionally classified based on the timing of onset of disease in relation to birth. Early-onset neonatal sepsis (EOS) occurs within the first 3 days after birth, due to vertical transmission of pathogens from mother to child. Late-onset neonatal sepsis (LOS) is defined as an infection which develops after day 3 of birth. For both EOS and LOS, the incidence and severity increases with decreasing gestational age, with very-low birth weight and preterm infants being most at risk for severe sepsis [2,5].

The heterogeneity of the disease makes early diagnosis of neonatal sepsis challenging. Blood culture remains the gold standard [6], but this requires adequate volumes of blood samples and is linked to underdiagnosis in neonates [7]. Moreover, currently used biomarkers, such as C-reactive protein, interleukin-6 (IL-6), and procalcitonin, show low discriminative value for diagnosis [2,8,9]. As the disease burden is high, lack of diagnostic tools generally leads to immediate administration of broad spectrum antibiotics when infection is suspected, resulting in overuse of antimicrobials in noninfected patients. It remains, therefore, of utmost importance to identify novel biomarkers to improve accurate and timely diagnosis of neonatal sepsis.

Variables relating both to the invading pathogen as well as to the ability of the neonate to mount an infection influence the outcome of neonatal sepsis. Current treatment of neonatal sepsis is, however, limited to antimicrobial therapy and supportive care, leaving the dysregulated immune response largely untreated. New targets of the dysregulated immune system and drugs to modulate these targets are therefore warranted.

Preclinical animal models are invaluable in biomedical research and drug development, but available models have so far not yielded new diagnostic biomarkers or novel treatment targets that significantly improve the outcome of neonatal sepsis [10]. Therefore, new preclinical models that can complement the currently available arsenal of models to study neonatal sepsis and pharmacological interventions are needed. For this, we propose zebrafish (*Danio rerio*) larvae.

**Fig. 1.**
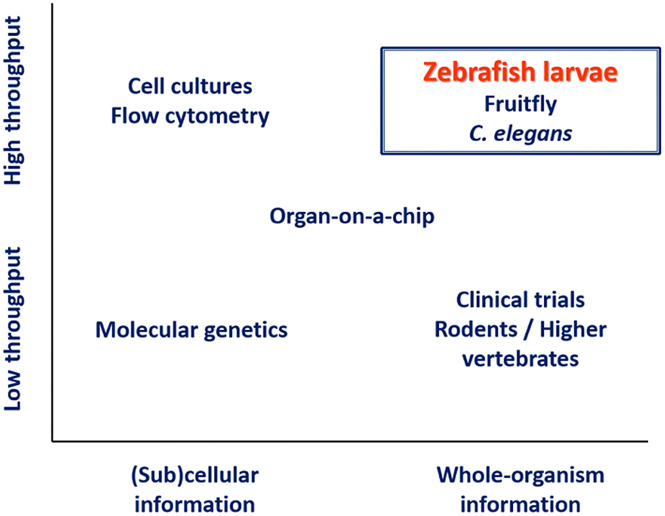
Schematic overview of available methods for biomedical research. Of the species that can bridge the gap between high-throughput *in vitro* methods and low-throughput animal and human experiments, zebrafish larvae have the advantage of being immunologically highly similar to humans. Adapted from Schulthess *et al.* [13].

Zebrafish larvae are being extensively used in biomedical research due to 70% genetic homology to humans, high reproductive capacity, and genetic tractability [11]. At larval stages, the small size and optical transparency [12] make zebrafish larvae a powerful whole-organism preclinical model with high-throughput potential. As such, these larvae can bridge the gap between high-throughput *in vitro* methods and low-throughput animal and human experiments, as illustrated in Fig. 1 [13]. Their potential for studies on neonatal sepsis is supported by the fact that zebrafish exhibit an immune system that is remarkably similar to humans, both in terms of types of immune cells and signaling pathways [14–16] and by the fact that the developmental state of the larval immune system is highly similar to human neonates [17].

This review addresses the applicability of zebrafish larvae as a preclinical model organism to study neonatal sepsis. Hallmarks of the immune system of both species are compared, with a specific focus on early life. For zebrafish larvae, the first 5 days post-fertilization are considered, as during this time the ethical constraints according to European law are minimal, thereby capitalizing on their high-throughput potential. Additionally, studies in zebrafish larvae with relevant pathogens are reviewed, and the potential advantages and disadvantages of zebrafish larvae to address some of the current challenges in neonatal sepsis research are discussed.

## Developing Immune System

Human neonates primarily rely on the nonspecific innate immune system, including the complement cascade and lymphocytes (natural killer cells (NK)), monocytes (macrophages and dendritic cells), innate lymphoid cells (ILCs), and granulocytes (neutrophils). Monocytes, macrophages, and neutrophils appear during the first or second trimester of gestation [18]; however, these cells are relatively immature at birth with limited functionality compared to adult’s innate immune cells. The innate immune system closely interacts with the adaptive immune system (T-cells and B-cells), which is instructed through antigen presentation cells (monocytes, macrophages, and dendritic cells), but this pathway is still developing in neonates and skewed toward anti-inflammatory and tolerogenic responses [19,20].

The innate immune system is also the first to develop in zebrafish larvae, being fully functional at 2 days post-fertilization (dpf) [21]. The main cells in the innate immune system of zebrafish larvae are neutrophils and macrophages. The first macrophages can be detected at 24 hours post-fertilization (hpf) [22] and neutrophils at 48 hpf [21]. Similar to human neonates, in the first 5 days post-fertilization, the adaptive immune system is still functionally inactive.

**Fig. 2.**
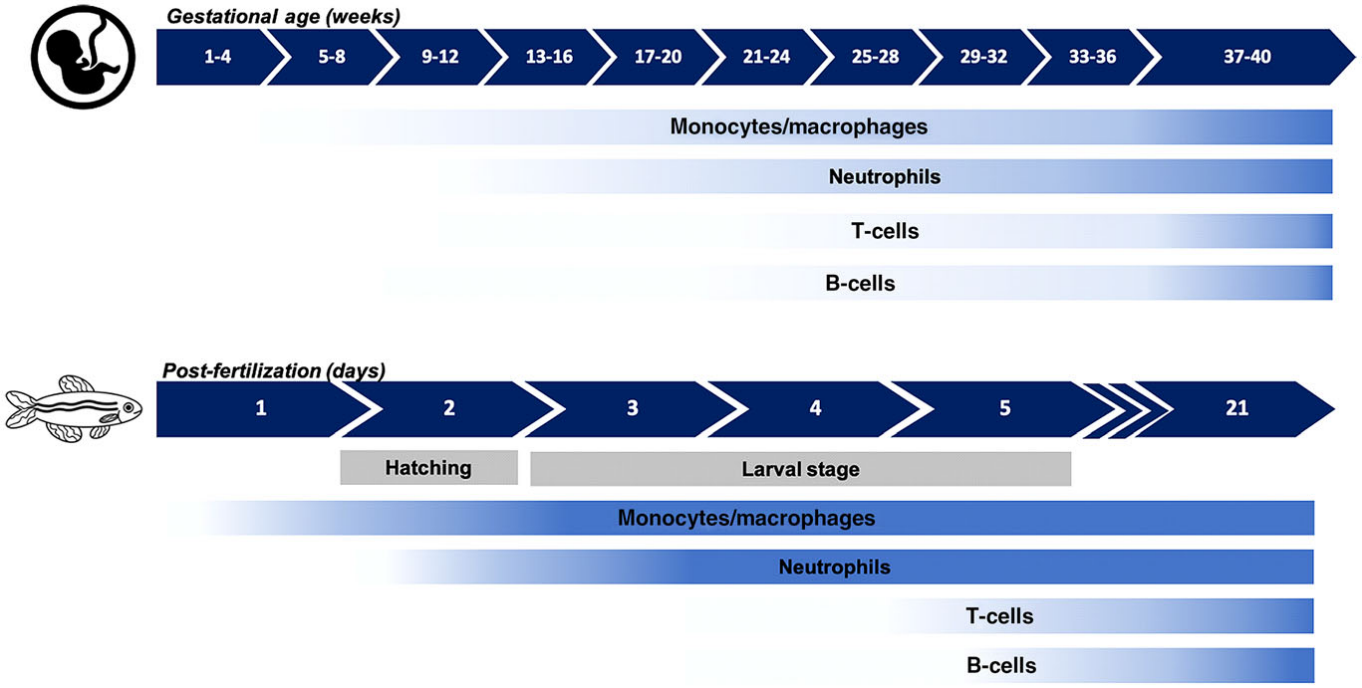
Schematic representation of the development of functional immunological cell types during gestation and development of human fetuses and zebrafish larvae.

Fig. 2 provides an overview of the development of important cell types in the immune system of humans and zebrafish. The following sections describe immunopathology of neonatal sepsis and compare the innate immune system of both humans and zebrafish more in detail.

### Immunopathology of Neonatal Sepsis

Sepsis is initiated by the immune system in response to a pathogen. In adults, increasing evidence illustrates two main immune hallmarks: sustained hyperinflammation and subsequent immune suppression. Both phases are initiated at the onset of infection and may alternate and occur at variable times during a sepsis episode. Hyperinflammation is characterized by the release of cytokines and pro-inflammatory mediators, a process known as “cytokine storm.” This unbalanced inflammatory response also includes the activation of the complement system, the coagulation cascade, and endothelial cells. The massive release of pro-inflammatory molecules and the uncontrolled activation of the complement system cause tissue damage and organ dysfunction, while the consumption of coagulation factors and platelets leads to hemorrhages, all of which can be fatal. In an attempt to return to immune homeostasis, several anti-inflammatory molecules are released, leading to a hypo-responsive immune state, the immune suppression phase [23]. In this phase, a patient may be unable to control the infection and is susceptible to new opportunistic infections [24], both of which could lead to death. These mechanisms are only partly understood in neonates. Studies using neonatal cord blood showed that the neonatal immune system exhibits different immune responses to pathogens compared to adults. It is thought that both the severity and high mortality in the acute phase of neonatal sepsis seem to be caused by a dysregulation of the neonatal pro-inflammatory immune response [25]. However, recent studies revealed that both pro-inflammatory and hypo-inflammatory responses are present at the onset of LOS, with elevated IL-6 and IL-10 levels and elevated Il10/tumor necrosis factor alpha (TNFα) ratios compared to noninfected neonates (LOS) [26,27]. Moreover, host–pathogen interactions may vary over time within a patient and between patients and are affected by several factors including gestational and postnatal age and the causative pathogen [25].

### Complement System

The complement system comprises over 30 proteins that induce inflammatory responses and improve bacterial opsonization [28]. Moreover, it modulates the adaptive immune response. In humans, synthesis of complement factors (C proteins) starts around week 5 of gestation, but the system does not reach its full capacity until 12–18 months after birth, leaving all major factors to be decreased in neonates, especially in premature neonates (<34 weeks GA) [29].

Genes encoding the principal components of the complement system have been identified in zebrafish [30]. Although not all components of the complement system have been functionally characterized in zebrafish, the C1q proteins, central to the classical complement activation pathway, exhibit expressional [31] and functional [32] similarities to higher vertebrates.

In zebrafish larvae, complement components are maternally transferred both in the form of protein and mRNA. These components then play a central role in protecting the externally fertilized embryo from pathogenic attacks at the earliest stages of development, before the cellular parts of the innate immune system have developed [30]. From 3 to 5 days post-fertilization, well after the maternal to zygotic shift, complement factors have been found to be expressionally induced in the embryo by different pro-inflammatory stimuli [33,34], indicating the complement system to be an active part of zebrafish’s innate immune system at this stage. However, at least in the case of the reaction to lipopolysaccharide (LPS) stimulation, transcriptional upregulation of the complement system is not the dominant immune response [31]. Informative zebrafish experiments to investigate functions of the complement system can be constructed with due consideration given to the certainty of homologous functionality of specific components. It is important to understand that at early stages of zebrafish embryonic development, the complement system occupies a central role that is not mirrored in neonatal development. These fundamental differences should be kept in mind when experiments are designed and results about the relative importance of complement components in the overall immune responses are interpreted.

### Natural Killer Cells

NK cells are cytotoxic cells that induce apoptosis through the release of granzymes (performin, granzyme B). Additionally, they mediate protection of the host by secretion of cytokines and chemokines, among others interferon gamma (IFNγ), which, in its turn, activates the adaptive immune system. Fetal NK cells appear around week 9 of gestation and are present in higher counts through gestation and at birth compared to adulthood [35]. However, the cytotoxic activity of neonatal NK cells is decreased compared to adults, mainly due to a low activity of the CD56dim cells in neonates [36].

In zebrafish, NK cells have been identified in adult tissues and appear similar to mammalian NK cells in terms of surface receptor repertoire [37]. Genetic and biochemical approaches have revealed more variety of NK lysins, one class among several of the bactericidal peptides stored in cytoplasmic granules in NK cells, in zebrafish compared to mammals [38]. However, the temporal emergence of zebrafish NK cells has not yet been established, before this is established the use of zebrafish larvae in studies on NK cells and immune response processes they mediate, is not warranted.

### Innate Lymphoid Cells

ILCs are lineage-negative lymphoid cells that mediate inflammatory and anti-inflammatory responses. Like NK cells, ILCs are part of the innate immune system and do not express antigen receptors. ILCs activate the acquired immune system by releasing cytokines such as IFNγ and TNFα and have been detected in human fetal material with the highest counts being present in the second trimester of pregnancy [39,40].

All three types of ILC have been identified in adult zebrafish based on their ability to express similar repertoires of cytokines, including elevated expression of IFNγ and TNFα upon bacterial challenge [41]. Like NK cells, the emergence of ILCs in embryonic development is hitherto unresolved, which should be kept in mind when selecting an experimental model to study ILC functions.

### Neutrophils

Neutrophils are a key component of the innate immune system and the most abundant type of leukocytes. Mature neutrophils appear around week 16 of gestation and are present in lesser concentrations in neonates compared to adults [42]. This is because neonatal bone marrow is deficient in producing neutrophil progenitor cells, and the neonatal neutrophil storage pools are reduced compared to those of adults, increasing the risk for neutropenia [43]. Apart from quantitative deficiencies, neutrophils in preterm neonates between 28 and 36 weeks GA show functional deficiencies with decreased phagocytic function, decreased chemotaxis [44], and impaired neutrophil extracellular trap (NET) formation [45].

Functionally mature neutrophils develop in zebrafish at 48 hpf [46]. An array of tools, such as fluorescent reporter lines, knockout mutant lines, and standardized assays, have been developed to study neutrophil maturation and behavior [47–49]. Such tools have been applied to address fundamental questions regarding neutrophil chemotaxis and reactivity to cytokine stimulation and their role in the resolution of inflammation and NET formation [48–51]. These studies have highlighted the role of neutrophils in initiation of inflammation and in resolution of inflammation and provided important insights in the regulatory mechanisms that underlie their role in the innate immune response and tissue homeostasis [52]. As such, zebrafish larvae appear to be an appropriate preclinical model to evaluate the role of neutrophils in inflammation and sepsis.

### Antigen-presenting Cells

Antigen-presenting cells (APCs) include monocytes, dendritic cells, and macrophages that present antigens through major histocompatibility complex (MHC) to T-cells, thereby activating the acquired immune system. APCs appear around week 12 of gestation in the thymus and lymph nodes [53]. However, in neonates, APCs are present in lower amounts compared to adults and the expression of MHC class II on neonatal APCs, needed for a proper immune response, is decreased. It has been reported that monocytes from septic neonates express even lower levels of MHC class II compared to monocytes of non-septic neonates [54].

The most well-studied APCs in zebrafish are macrophages, which have been characterized in terms of development and functional maturation and in terms of their reaction to cytokines and pathogen stimulation. Phagocytically active zebrafish macrophages emerge at 1 dpf from the lateral plate mesoderm [22,46,55], and in many studies these have been found to be among the first responding immune cell types reacting to various bacterial and fungal pathogens [56–58]. Using combinations of fluorescent reporter lines, M1 and M2 activation status can be conveniently assessed by live microscopy [59]. Dendritic cells exhibiting MHC class II expression that have the capacity to activate T lymphocytes have been identified in adult tissues [60] and they are enriched in gut and skin [61] indicating their functional conservation. It is, however, uncertain when mature dendritic cells emerge during zebrafish development. As a result, zebrafish larvae may be suitable to study macrophage actions, but uncertainty remains about its suitability to serve as a model in the study of processes involving dendritic cells.

### Pattern Recognition Receptor

Recognition of invading pathogens is achieved through activation of pattern recognition receptors (PRRs). PPRs detect conserved microbial structures called pathogen-associated molecular patterns (PAMPs) or damage/danger-associated molecular patterns (DAMPs). Those microbial structures include DNA, lipoproteins, carbohydrates, and other structures. LPS is a PAMP, found on the cell surface of gram-negative bacteria. The most studied PRRs are the toll-like receptors (TLRs) through which PAMPs trigger a signal cascade that leads to the release of pro-inflammatory mediators that help control pathogens [62–64], although in animal models like the zebrafish, TLRs have also been shown to have an anti-inflammatory immune-regulatory function [65,66]. There are 10 TLRs in humans recognizing different DAMPs or PAMPs [67]. Tlr4, which recognizes LPS, has received most attention regarding its role in sepsis [68], although several other TLRs are being investigated as possible targets for therapeutic intervention in sepsis treatment; Tlr2 and Tlr4 primarily through antagonistic anti-inflammatory mechanisms and other TLRs through agonistic mechanisms to enhance the immune response to infections [69]. Recent findings that TLRs such as TLR2 also play an important role in negative control of inflammatory processes [66] may indicate that agonistic activation could also be beneficial to suppress hyperinflammatory responses under certain circumstances. It has been shown that Tlr4 expression is reduced in neonates, especially those with very low birth weight [70].

Considerable research efforts in the past decades showed similarities between PRRs and intracellular signaling pathways to be extensive between humans and zebrafish. Homologs of most of the human TLRs have been identified in zebrafish [67]. Functional analyses have established that the accessory adaptor molecule Myd88 occupies the same central position in the intracellular signaling cascades downstream of all TLRs except TLR3 [71,72]. However, while the similarities between human and zebrafish responses to bacterial infection and PAMP stimulation are striking, it should be noted that important differences and sometimes conflicting observations have been reported, particularly regarding the molecular pathways involved in LPS signaling. Two *in vitro* studies have found that while zebrafish embryos do mount a clear inflammatory response to LPS stimulation, it does not appear to be mediated through Tlr4 [73,74]. The notion that zebrafish Tlr4 does not recognize LPS has recently been challenged, with the identification of a zebrafish gene encoding the coreceptor myeloid differentiation factor-2 (Md-2) [75], suggesting the mechanisms of LPS signaling in zebrafish may be more similar to those in humans after all. Conflicting observations have been made regarding the role of Myd88, specifically in LPS signaling, as an *in vitro* study found the zebrafish inflammatory response to LPS to be independent of Myd88 [73], while an *in vivo* mutant study reported the opposite [72]. Considering that some disease models are based on LPS stimulation [76,77], caution should be exercised when drawing conclusions regarding the exact nature of LPS-mediated signaling, even if the inflammatory responses caused by this stimulation may still serve as a model for certain aspects of sepsis research.

### Cytokine Production

Both pro- and anti-inflammatory cytokines are crucial for cell signaling, and initiation, maintenance, and resolution of host responses to infections. Several pro-inflammatory cytokines can be used as diagnostic markers for sepsis, including IL-1β, IL-6, IL-8, IL-23, TNFα, and IFNγ. Reviewing the potential of each biomarker in the diagnosis of neonatal sepsis is beyond the scope of this review. Neonates with sepsis present with elevated levels of circulating cytokines [25]; however, gestational age does influence cytokine responses and very preterm neonates show reduced or altered cytokine production in response to sepsis, possibly explaining their higher risk for severe infection [78–80].

**Fig. 3.**
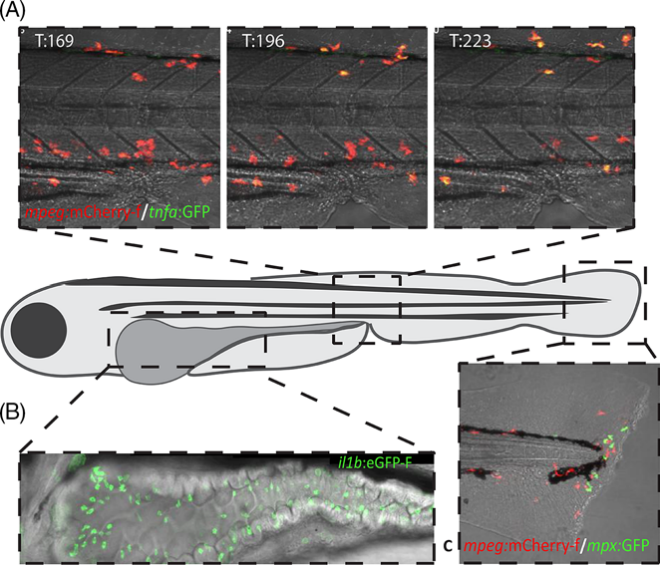
Examples of applications of fluorescent reporter lines in zebrafish larvae. A: Three still images from a confocal timelapse microscopy video showing the gradually increased expression of *tnfa* by macrophages after infection with *E. coli* via the duct of Couvier at 3 DPF, in the Tg*(mpeg1*:mCherry-F)^ump2^ [57] fishline, with macrophages expressing red fluorescent mCherry, crossed with the Tg(*tnfa*:eGFP-F)^ump5^ [59]. The time (in minutes) after infection is indicated in the upper left-hand corner of each image. The overlap of red and green fluorescent signal makes *tnfa* expressing macrophages appear yellow. B: A single confocal stack showing intestinal epithelial cells expressing *il1b* in the TG(*il1b:*eGFP-F)^ump3^ [57] reporter line, after intestinal colonization by unspecified commensal microbes. C: Stereo-fluorescent microscopy image in the Tg*(mpeg1*:mCherry-F)^ump2^/TG(*mpx:*GFP)^il14^ [47,57], showing red fluorescent macrophages and green fluorescent neutrophils migrating to a site of injury in a widely applied tailfin amputation assay.

Zebrafish have been used extensively in cytokine research and the pro-inflammatory cytokines that drive sepsis have all been identified, including IL-8 [50], which is absent in mice and rats. Transcription levels of IL-1β, TNFα, INFγ, IL-6, and IL-8 have been found to follow similar temporal profiles of transcriptional induction and subsequent return to baseline upon inflammatory challenges such as LPS injection and tissue amputation. All exhibited robust induction within the first 12 hours after challenge and rapid resolution [81–83], indicating good conservation of their roles in mediating early responses to inflammatory stimuli. In zebrafish, fluorescent lines have been generated for IL-1β [57], TNFα [59], and IFNγ [84] enabling for instance *in vivo* microscopy approaches as illustrated in Fig. 3. IL-10 has been assessed as a marker of anti-inflammatory signaling and alternative macrophage activation (M2) in numerous studies [85,86]. It exhibits transcriptional induction subsequent to the inflammatory response to LPS stimulation [83].

## Zebrafish Larvae as Model for Neonatal Sepsis

Animal models have provided insight into the pathogenesis of neonatal sepsis; however, this has hitherto not lead to considerable improvements in available treatment strategies [10]. This may be because of the absence of clinically relevant features in the current preclinical models [87], due to discrepancies in pharmacological effects between traditional preclinical species and humans, or because timing and dosage are essential aspects of successful sepsis treatment [88–90]. Moreover, traditional animal models have limited high-throughput potential. Keeping in mind the known differences between neonates and zebrafish larvae and the knowledge gaps in zebrafish larvae described in the previous section, zebrafish larvae may provide a useful complementary preclinical model to overcome some of these shortcomings. Moreover, the different entities of neonatal sepsis (e.g. EOS versus LOS) and specific causative pathogens influencing the host–pathogen interaction have to be recognized. In the first 5 days post-fertilization, zebrafish larvae may best reflect scenarios of EOS.

As with any preclinical model, it is important to be conscious about known differences between neonates and zebrafish larvae and the knowledge gaps in zebrafish larvae described before. For example, the well-established similarities of TLR signaling [67,91] and key important cytokine responses between humans and zebrafish means initiation and dynamics of pro-inflammatory signaling can be studied in zebrafish embryos, with the caveat that LPS signaling through TLR4 is not entirely resolved [73–75]. Thus, to model pathogen recognition in EOS caused by *E. coli*, it may be advisable to use live bacteria, or at least a more complex pro-inflammatory stimulus than LPS. For group B *Streptococcus* (GBS) on the other hand, it may be possible to use purified PAMPs to investigate inflammatory initiation, since the Tlr2 signaling appears to be more similar between humans and zebrafish [91].

In the next paragraph, we will discuss studies on pathogens that cause neonatal sepsis. Major findings are summarized in Table 1.

**Table 1.** Overview of major findings obtained in zebrafish larvae on infections with pathogens relevant for neonatal sepsis with indications of methods of infection. (SI: systemic infection; LI: localized infection; FB: food-borne).

Pathogen	Major findings	References
*Streptococcus agalactiae (GBS)*	- Serotype-dependent GBS virulence.	[92]^*SI*^
	- Upregulation of interleukin-1β and interleukin-8 genes, related to neutrophil activation and recruitment.	
	- Systemic infection leading to blood–brain barrier crossing infection involving host transcriptional suppressor Snail1	[93]^*SI*^
*Escherichia coli*	- Flagellar serotype linked to virulence, pathogenic manifestations, for example, epithelial protrusions in the tail and trunk and temporal window of opportunity for pharmaceutical intervention.	[94]^*SI*^
	- Importance of locus of enterocyte effacement (LEE) type 3 secretion system to intestinal virulence of enterohemorrhagic *E. coli* (EHEC).	[95]^*FB*^
	- Rapid but brief *il1b* expression from macrophages versus delayed but sustained *il1b* expression from neutrophils.	[57]^*LI*^
	- Degranulation and bactericidal activity of neutrophils which are not in direct contact with bacteria.	[96]^*LI*^
	- Mutant library screening of an extraintestinal pathogenic *E. coli* (ExPEC) to investigate the virulence gene repertoire *in vivo*.	[97]^*SI*^
*Staphylococcus aureus*	- An intracellular niche in neutrophils is a critical bottleneck leading to clonal expansion of single strains after multistrain infection.	[98]^*SI*^
	- Microtubule-associated protein 1 light chain 3 (Map1lc3) associates with *S. aureus* upon neutrophil phagocytosis and provides an intracellular niche which enhances survival of the pathogen.	[99]^*SI*^
	- Subcurative dosages of antibiotics support preferential clonal expansion of resistant strains in mixed-strain infection *in vivo.*	[100]^*SI*^
	- Protective neutrophil activating role for nerve growth factor *b* (Ngfb) and its receptor tropomyosin-related kinase receptor A (Trka)	[101]^*SI*^
	- Identified a metabolic adaptation strategy in the pathogen to achieve daptomycine resistance and evading neutrophil chemotaxis.	[102]^*LI*^
*Staphylococcus epidermidis*	- High-throughput adaptations of injection strategies and analysis of infectious burden. Transcriptomics data from multiple time points of infection.	[103,104]^*SI*^

### Infection Models in Zebrafish Larvae with Pathogens Relevant for Neonatal Sepsis

Zebrafish larvae are often utilized to study infections and host–pathogen interactions. The most common approach involves induction of infection through microinjection into specific sites to create systemic or localized infections, followed by microscopy-based or transcriptional assessment of host responses [57,58]. Systemic infection models are used for the assessment of overall transcriptional responses in the search for biomarkers [99, 103, 105], while localized infections are used for highly detailed studies of interactions between pathogens and immune cells [58,106]. Alternatively, the ease of injection in zebrafish larvae can be leveraged to generate pathogenic screening tools where mutant libraries of pathogenic strains are evaluated in a vertebrate host, in search for novel virulence factors [97], to investigate pathogen community dynamics [98,100], or to test properties of mutant strains *in vivo* in an immunocompetent vertebrate [102,107]. Finally, infections have also been induced through the intestinal route, either food-borne or by keeping larvae in pathogen-containing incubation medium [95,108]. Although in the first 5 days post-fertilization zebrafish larvae may best reflect EOS, pathogens related to LOS are also discussed.

#### Streptococcus Agalactiae

*S. agalactiae* is a gram-positive bacterium belonging to GBS [109] and is one of the main causes of EOS [110]. *S. agalactiae* has been used in zebrafish larvae of 3 dpf to study pathogen and host factors that are essential for the progression of sepsis [92]. The results showed upregulation of the pro-inflammatory cytokines IL-1ß and IL-8, related to neutrophil activation and recruitment, which is also a key characteristic of the GBS infection found in a mouse model of meningitis [50,111]. Additionally, capsule and anchored lipoteichoic acid were identified as virulent factors for *S. agalactiae* infections [92], validating results found in a rat model of GBS infection [112] and in *in vitro* studies with human cell lines [113].

The optical transparency of the zebrafish embryo was used to demonstrate that *S. agalactiae* is able to cross the blood–brain barrier [92]. This may suggest zebrafish larvae could be a useful model to study localization and spread of infections throughout the body, including the brain.

#### Escherichia Coli

*E. coli* is also one of the main pathogens of neonatal sepsis, causing both EOS and LOS [114]. Barber *et al*. [94] provide an example of the versatility of zebrafish larvae to study infections. They tested different extraintestinal pathogenic *E. coli* (ExPEC) strains and measured whole-organism transcriptomics and various pathological endpoints and evaluated antibiotic treatment regimens. The larvae exhibited symptoms observed in neonatal sepsis, including cytokine storm, tachycardia, edema, and vascular leakage [2]. Strain differences in flagellar serotype and flagellin levels were shown to correlate with differences in pathological development and transcriptional profiles of cytokines. Furthermore, strain differences were evident in the efficacy of antibiotic treatments and the impact of early versus delayed antibiotic treatment initiation. This illustrates that zebrafish larvae allow for the temporal evaluation of infection development and treatment outcome.

#### Staphylococcus Aureus

*S. aureus* is a gram-positive species that is highly adaptable and can colonize virtually any host tissue, causing infections from skin abscesses to bloodstream infections that lead to LOS [115].

Studies in zebrafish larvae infected with *S. aureus* support the hypothesis that phagocytes act as “Trojan horses” for this pathogen. The optical transparency of the larvae and ease of genetic manipulation allowed for the identification of intracellular niches of *S. aureus* in phagocytes, specifically in neutrophils, that serve as reservoirs protecting the pathogen from immune destruction and that ultimately release massive amounts of pathogens that will lead to systemic infections [98]. This study identified this intracellular niche as a novel target for treatments of *S. aureus* infections, illustrating how zebrafish larvae can provide key information that can lead to new therapeutic approaches.

#### Staphylococcus Epidermidis

*S. epidermidis* is an opportunistic pathogen that can cause LOS [116]. This member of the CONS family is naturally present in human skin lesions where it is generally harmless. CONS can be pathogenic in preterm neonates due to their relative immature immune system and the high number of invasive medical procedures performed in this population increases their risk of infection [117].

Veneman *et al.* established a protocol for *S. epidermidis* infections in zebrafish larvae [103]. Their study revealed genes involved in the pathogenesis of *S. epidermidis* infections, such as *mfap4* which is related to cell adhesion. Moreover, the protocol has high-throughput capabilities and allows for automated fluorescence-based quantification of the infection and transcriptomic analysis. This application of high-throughput concepts supports the broad potential of this species in future research on the diagnosis and treatment of neonatal sepsis.

The high-throughput potential has also been illustrated by Philip *et al.* who used LPS to induce sepsis symptoms like vascular leakage, exudative edema, extravasation of neutrophils, alterations in the coagulation system, immune activation, and production of reactive oxygen species [76]. Subsequently, they screened a library of 96 small molecules targeting epigenetic and immune modulators for efficacy regarding these endpoints, taking advantage of the larval optical transparency. This study identified promising chemical entities including Fasudil, known to be effective in treating vascular leakage in a murine model of sepsis, thereby supporting the intra-species scaling potential of findings in zebrafish larvae.

### Limitations of Zebrafish Larvae as Model for Neonatal Sepsis

Despite the known similarities in the immune response of zebrafish and humans, particularly, the exact emergence of certain components of the innate immune system, such as NK cells and ILCs in zebrafish larvae, remains unknown. In addition to that, uncertainties about the exact mechanism of LPS signaling in zebrafish larvae may impact translatability of findings on infections with gram-negative bacteria.

An intrinsic limitation of sepsis research in zebrafish larvae is that human pathogens infect and grow at 37°C, while the optimal temperature for the maintenance of zebrafish and their larvae is 28°C [118]. This temperature could lead to attenuated activity of human pathogens and as a consequence pathogen infections in zebrafish larvae might not accurately reflect infections in humans. This can be (partially) overcome by slightly adapting the maintenance temperature of the larvae to 31°C [119] as a compromise suitable for both pathogen and host or by using related pathogens that can infect zebrafish at that lower temperatures [120].

Also, the scarcity of monoclonal antibodies against zebrafish’s cell surface markers limits the use of common molecular biology techniques like immunohistochemical staining or flow cytometry. However, it can be anticipated that the increasing popularity of the zebrafish and its larvae will lead to a wider range of monoclonal antibodies against zebrafish antigens becoming available.

Contrary to higher vertebrate species, the internal exposure of drugs in pharmacological or toxicological studies or screens is currently hardly ever quantified in zebrafish larvae and drug concentrations in the surrounding medium are often unjustly used as a proxy for drug exposure. Without adequate quantification of internal drug exposure, interpretation of observed effects (or lack thereof) is limited, which may lead to false-negative findings for drug efficacy in this species. Progress is, however, being made in the development of novel methods that allow for the quantification of internal drug exposure in zebrafish larvae [121,122].

## Future Perspectives

Successful treatment of neonatal sepsis requires 1) the discovery of specific and predictive biomarkers for diagnosis and evaluation of treatment response and 2) the identification of novel treatment targets and effective drugs for these targets. Giving its unique features, the zebrafish larva is a promising preclinical model that can complement the available methods for research in both areas.

The discovery of disease-specific biomarkers for neonatal sepsis is essential for timely initiation and cessation of treatments, ensuring optimal efficacy and minimizing the development of resistance. Furthermore, biomarkers are required for patient selection in trials evaluating new therapies, as inclusion of misdiagnosed patients or the inability to stratify patients into subgroups that would benefit from targeted therapy will reduce the statistical power to detect drug effects.

A key advantage of zebrafish larvae in biomarker discovery is their suitability for hypothesis-generating whole-organism transcriptomic, proteomic, and metabolomic studies. Omics techniques may provide novel information about the pathophysiology of sepsis and identify new diagnostic biomarkers. Due to the disease complexity, it is unlikely that a single biomarker could serve as a diagnostic marker for neonatal sepsis; however, the combination of high-throughput data acquisition and advanced analysis techniques for large datasets could lead to the identification of diagnostic fingerprints that are composed of multiple markers.

Regarding novel treatment targets, research in sepsis therapies has in recent years been focused on agents to control the exacerbated inflammatory responses [123]. The demonstrated similarities in the (temporal) hallmarks of infections in zebrafish larvae and the ease of use of this whole-organism model in high-throughput screening studies may expedite the discovery of novel targets and identification of new drugs for targeted therapies that translate well to humans.

The translation of pharmacological findings in zebrafish larvae to human neonates may be improved by applying pharmacological modeling approaches. These approaches allow for quantitative interspecies scaling by correcting for known differences in (patho)physiology between species. This was recently illustrated in the field of tuberculosis, a disease that is studied in zebrafish larvae infected with *M. marinum,* a close relative of the human pathogen *M. tuberculosis*. By correcting for differences in drug sensitivity between the two bacterial species and differences in the growth phase of the bacterial infections in larvae and humans, findings in zebrafish larvae on the efficacy of isoniazid were successfully translated to humans [124]. Similar approaches could be applied in the research on neonatal sepsis to overcome potential issues arising from differences in maturation and function of immunological cell types or signaling pathways, or from differences in body temperature.

In recent years, manipulation of the gut microbiome, the communities of microbes in the intestine, through probiotics and prebiotics has shown potential as preventative strategy against neonatal sepsis [125]. Zebrafish larvae are uniquely suitable for studies aimed at evaluating health-promoting effects of microbial colonization [126], and the microbiome was recently found to impact innate immune regulation through transcriptional regulation of *myd88* [127]. The evaluation of health benefits of specific bacterial strains is likely to bring about new advances in affordable sepsis prevention, and zebrafish embryos are sure to continue to bring added benefits to existing research models.

## Conclusion

Zebrafish larvae have been successfully used to model infections with pathogens causing neonatal sepsis, capturing several hallmarks of the immunological and phenotypical pathophysiology and allowing the identification of host and pathogen factors necessary for the establishment and spread of the infection. The representation of important aspects of human infections in zebrafish larvae opens up the possibility to include this vertebrate model in preclinical research, to complement existing *in vitro* and *in vivo* models with high-throughput potential, which will stimulate biomedical and pharmacological research on neonatal sepsis. This research may hold important keys for the discovery of new biomarkers and novel treatment targets as well as for screening of targeted drug therapies.
